# Supplementary material for: Real-time reliable determination of binding kinetics of DNA hybridization using a multi-channel graphene biosensor
Source: Nat Commun. 2017 Mar 21;8:14902. doi: 10.1038/ncomms14902 (PMC5364407; doi:10.1038/ncomms14902)
Supplement: Supplementary Information — Supplementary figures, supplementary table and supplementary methods [file ncomms14902-s1.pdf]

Supplementary Methods

As shown in the schematic cross-section of G-FET and its equivalent electrical circuit (Fig. 2d), the gate capacitance of a G-FET is related to four parallel plate capacitors (C_{G1}, C_{G2}, C_{G3} and C_Q) connected in series. C_{G1}, C_{G2}, and C_{G3} denote the capacitance between graphene and solution, the capacitance of the DNA to solution, and the capacitance between Pt gate and solution, respectively. They are all formed due to electric double layers on the interfaces and called as the “geometrical" capacitances of the device. C_Q denotes the quantum capacitance of graphene associated with the finite density of states. Therefore, the total gate capacitance C is given by

$$C = \left(\frac{1}{C_{G1}} + \frac{1}{C_{G2}} + \frac{1}{C_{G3}} + \frac{1}{C_Q} \right)^{-1} \quad (1)$$

When analytes (target DNAs) dock on the surface of the transistor channel, the additional DNAs give rise to changes in charges (Δq) at the solution-graphene interface. These capacitors will produce variations in electrostatic potential and in turn shift V_{cnp} by

$$\Delta V_{cnp} = \frac{\Delta q}{C} = \left(\frac{1}{C_{G1}} + \frac{1}{C_{G2}} + \frac{1}{C_{G3}} + \frac{1}{C_Q} \right) \Delta q = \frac{\Delta q}{C_{G1}} + \frac{\Delta q}{C_{G2}} + \frac{\Delta q}{C_{G3}} + \frac{\Delta q}{C_Q} \quad (2)$$

The plate distance can be determined by the Debye length that is theoretically given by $d = 2ce^2/\varepsilon_0\varepsilon_r k_B T$, where T is the temperature, k_B is Boltzmann’s constant and c is the concentration of ions in the electrolyte. The Debye length is calculated to be ~7.3 nm in 0.01×PBS. From the model of the parallel plate capacitors, $C_{G1}= S_{graphene}\ \varepsilon_r\varepsilon_0/d_1$, $C_{G2}=S_{graphene}\ \varepsilon_r\varepsilon_0/d_2$, and $C_{G3}=S_{Pt}\varepsilon_r\varepsilon_0/d$, where S_{Pt} is the contact area between the electrolyte and the Pt electrode, $S_{graphene}$ is the contact area between the electrolyte and graphene monolayer, ε_0 is vacuum permittivity (8.85×10^{-12} F/m) and ε_r is the relative dielectric constant of water (80). The plate distance of C_{G1} (d_1) can be approximated as half height of the measured DNA pair (~3.4 nm). The plate distance of C_{G2} (d_2) can be approximated as Debye length subtracted by d_1 (~3.9 nm). The plate distance of C_{G3} can be estimated by the Debye length (~7.3 nm). Moreover, because the Debye length is longer than the DNA length in our case, the whole DNA can be detected without charge screening. Because S_{Pt} (~7.85×10^6 μm^2) >> $S_{graphene}$ (~4.05×10^3 μm^2) (See the caption in **Supplementary Figure 8b**), the third item $\Delta q/C_{G3}$ in Supplementary Eq. (2) (ΔV_{cnp} due to DNA hybridization from Pt electrode) is negligible, as in previous studies (*Phys. Rev. B* 2015, 91, 205413 and *P. Natl. Acad. Sci. USA*, 2011, 108, 13002). Thus, the total geometrical capacitance (C_{TG}) of the electrolyte is estimated at ~3.9×10^{-4} μF by

$$\frac{1}{C_{TG}} = \frac{1}{C_{G1}} + \frac{1}{C_{G2}}$$

The C_Q of the graphene channel is estimated at ~8.1×10^{-5} μF by $C_q S_{graphene}$. Here, C_q is quantum capacitance per unit area of ~2 μF cm^{-2} (*Nano Lett.* 2009, 9, 3318 and *Nat. Nanotechnol.* 2008, 3, 654). C_Q is comparable to C_{TG} and should be taken into

account. The charge changes from T20 with 20 nucleotides can be described as $\Delta q = 20neS_{graphene}$. Then, Supplementary Eq. (2) can be written as

$$\Delta V_{cnp} = \frac{\Delta q}{C} = \left(\frac{1}{C_{G1}} + \frac{1}{C_{G2}} + \frac{1}{C_{G3}} + \frac{1}{C_Q} \right) 20neS_{graphene} \quad (3)$$

Using the above model and ΔV_{cnp} of ~0.220 V with P20 addition, the probe density (n) of P20 in 0.01× PBS can be estimated to be ~1.140×10^{11} cm^{-2} from Supplementary Eq. (3).. Similarly, the estimated density of the hybridized DNA T20 was ~$1.052 \times 10^{11} cm^{-2}$ from ΔV_{cnp} of ~0.203 V. Thus, the hybridization efficiency of duplex formation can also be estimated to be ~92.3 %.

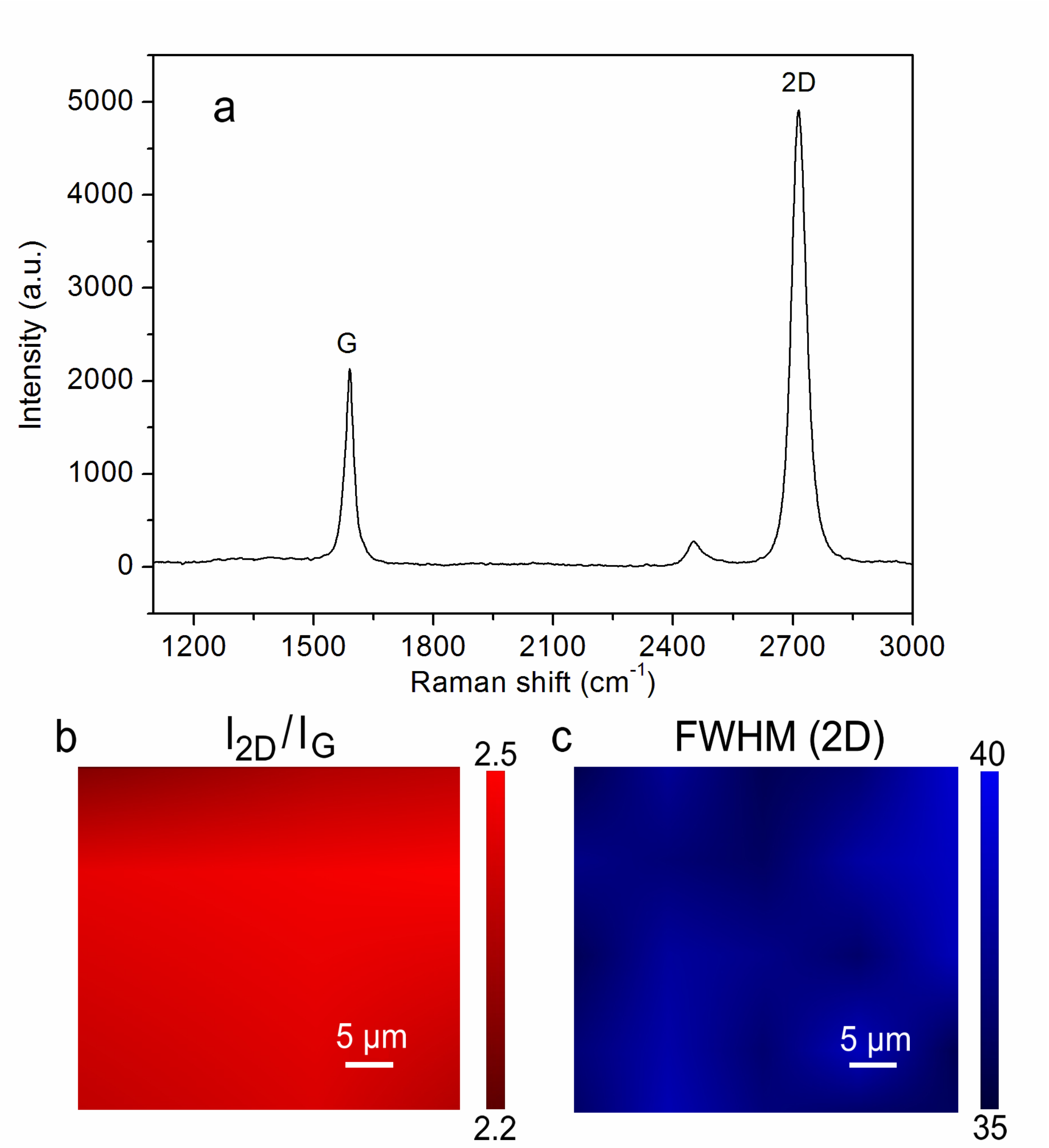

Supplementary Figure 1. Raman spectrum and Raman mapping of graphene used in the experiments. a, Raman spectrum of a graphene sample transferred onto a

SiO_2/Si substrate. **b,** Raman mapping of peak intensity ratios of I_{2D}/I_G. **c,** Raman mapping of full-width at half-maximum (FWHM) of 2D band. The Raman spectrum shows the typical features of monolayer graphene: (i) the intensity ratio $I_{2D}/I_G \geq 2$ and (ii) a single Lorentzian 2D band with a FWHM of 35–40 cm^{-1}.

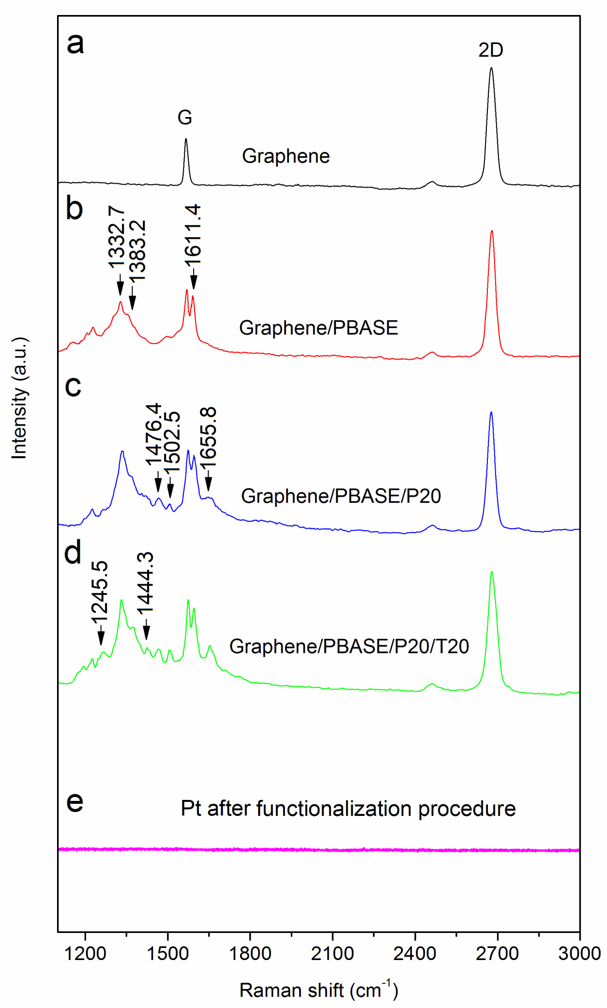

Supplementary Figure 2. Raman spectrum of graphene in the process of functionalization and hybridization. a, Bare graphene. **b,** Graphene/PBASE. **c,** Graphene/PBASE/probe DNA (P20). **d,** Graphene/PBASE/probe DNA (P20)/target DNA (T20). **e,** Raman spectrum obtained on Pt electrode after the whole functionalization procedure.

Raman spectroscopy was employed to study the graphene film after its interaction with PBASE, subsequently with probe DNA P20 and target DNA T20. The characteristic peaks of PABSE and DNA were clearly observed after addition of respective molecules. In low-frequency regions, several intrinsic signals due to PBASE molecules appeared. The peak at 1332.7 cm^{-1} was from sp^3 bonding. The peak at 1611.4cm^{-1} can be assigned to the pyrene group resonance and the peak at 1383.2cm^{-1} is due to the introduction of disorder arising from orbital hybridization of the molecule with the graphene plane. After the probe DNA or the target DNA was immobilized on the Graphene/PBASE layer, more peaks appeared, which can be assigned to the modes of DNA. After the whole functionalization procedure, neither PBASE nor DNA Raman signals were observed on the Pt electrode, indicating that the Pt was not functionalized in the process of graphene functionalization.

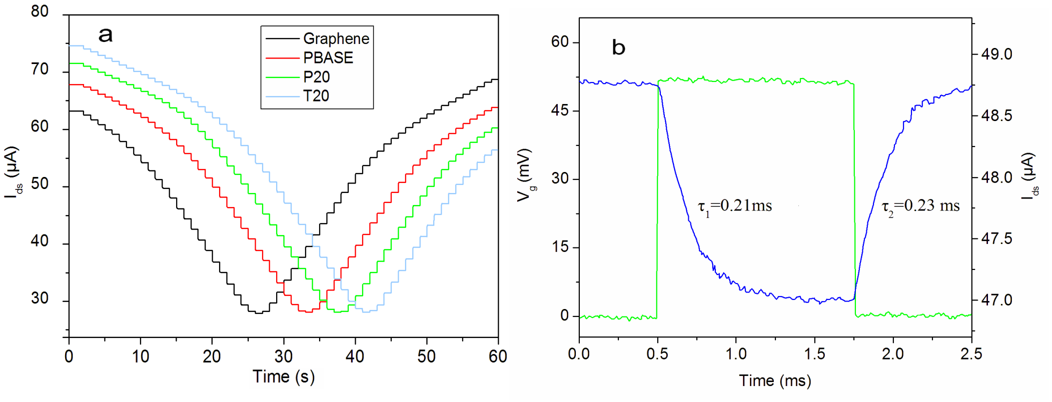

Supplementary Figure 3. a, Time dependence of I_{ds} as V_g varies from -1.2 to 1.7 V. **b,** Transient response of I_{ds} to V_g pulsed from 0 to 50 mV.

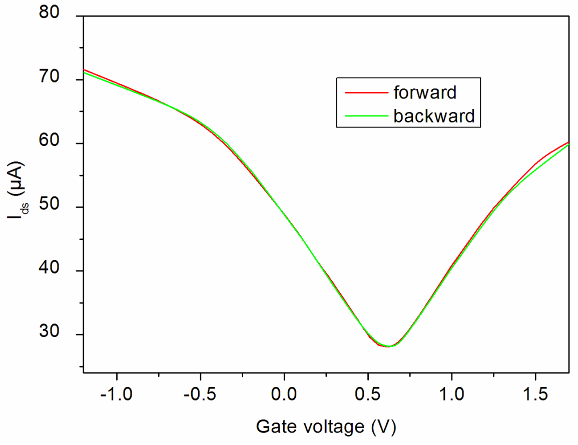

Supplementary Figure 4. V_g-I_{ds} transfer curve of the G-FETs with forward V_g (red) and backward V_g (green) sweeping in consecutive sweeps.

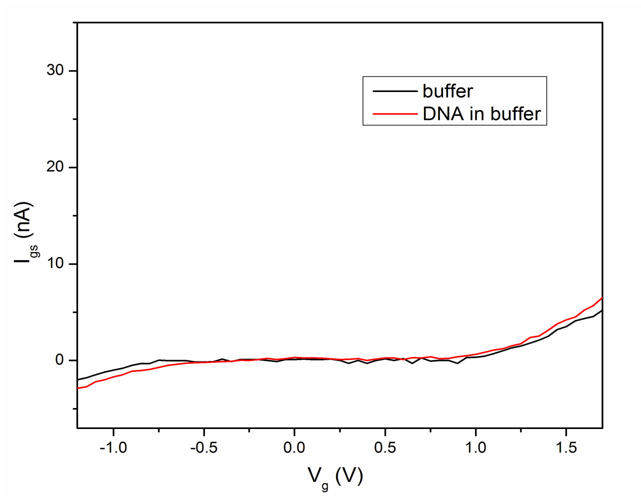

Supplementary Figure 5. Leakage current I_{gs} measured in buffer without DNA and in buffer with DNA as V_g sweeps from -1.2 to 1.7 V.

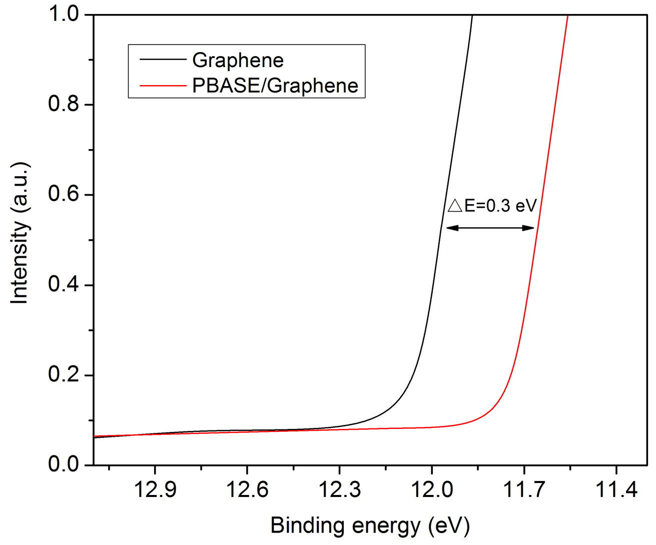

Supplementary Figure 6. Characterization of graphene before and after PBASE immobilization by ultraviolet photoelectron spectroscopy (UPS). UPS was used to record the change in work function induced by the adsorption of PBASE. -5V bias is applied to the substrate to overcome the work function of the analyser (~4.4 eV). The work function of the sample is calculated by the equation $\phi = h\nu - W - \Delta V$, where ν is the UV light energy (21.2 eV), W is the energy at secondary electron cut-off, and ΔV is the voltage bias. Consistent with the hole doping effect, the work function is increased by ~0.3 eV.

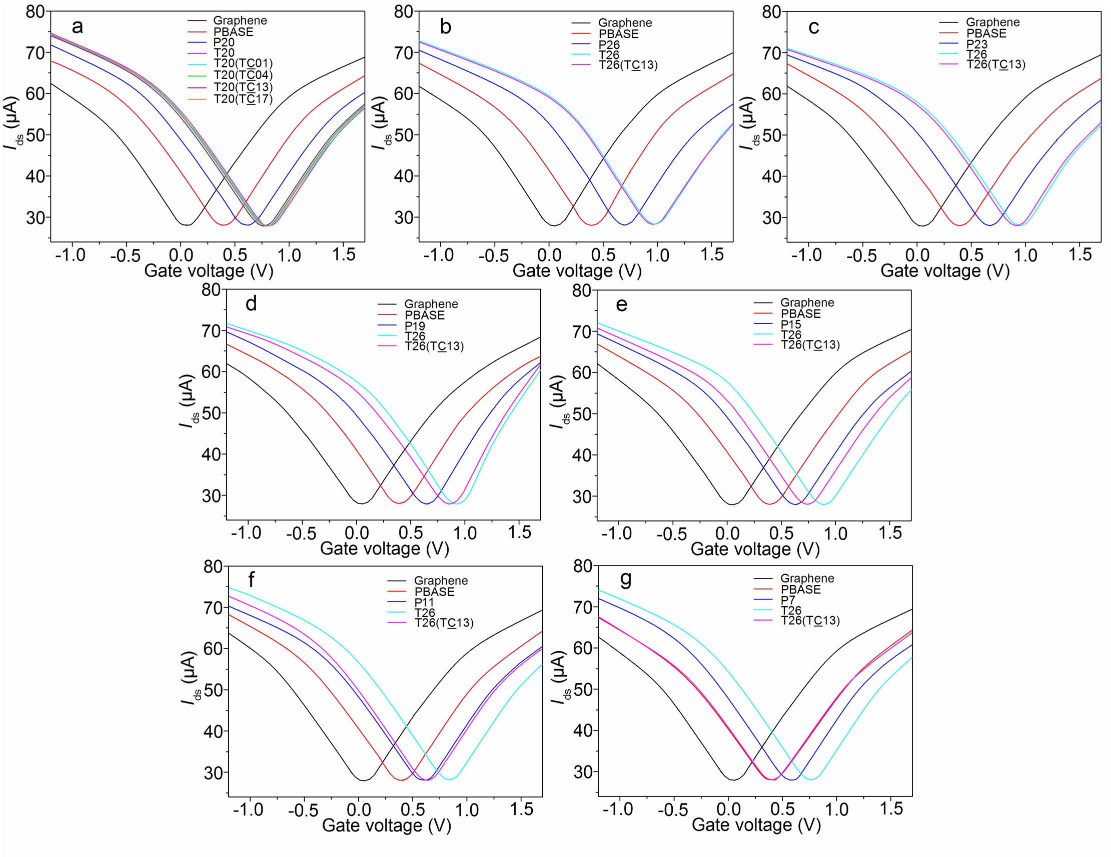

Supplementary Figure 7. Transfer curves for a G-FET device in response to target DNAs of different mutation locations and probe DNAs of different sizes. a, P20 immobilization, hybridization with T20 and its mutant T20(T**C**01) (T to C at position 1), T20(T**C**04), T20(T**C**13) and T20(T**C**17). **b,** P26 immobilization, hybridization with T26 and its mutant T26(T**C**13). **c,** P23 immobilization, hybridization with T26 and its mutant T26(T**C**13). **d,** P19 immobilization, hybridization with T26 and T26(T**C**13). **e,** P15 immobilization, hybridization with T26 and its mutant T26 (T**C**13). **f,** P11 immobilization, hybridization with T26 and its mutant T26 (T**C**13). **g,** P7 immobilization, hybridization with T26 and its mutant T26 (T**C**13). In all cases, the target DNA's concentration is 5 nM. These charge neutrality point voltages (ΔV_{cnp}) all shift in the positive gate voltage direction and the sizes of the shift depend on target DNA charges.

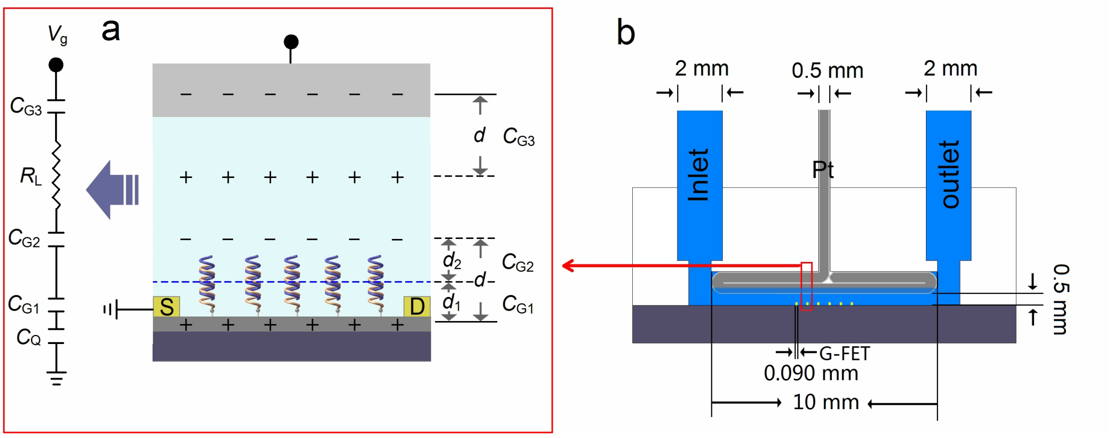

Supplementary Figure 8. a, Schematic diagram of the sensing model together with the equivalent circuit with four parallel plate capacitors (C_{G1}, C_{G2}, C_{G3}, and C_Q) and a resistance (R_L) connected in series. C_{G1}, C_{G2}, and C_{G3} denote the capacitance between graphene and solution, the capacitance of the DNA to solution, and the capacitance between Pt gate and solution, respectively. C_Q denotes the quantum capacitance of graphene associated with the finite density of states due to Pauli principle. R_L is the electrical resistance of the ionic solution. **b,** Schematic diagram of the location of Pt electrode within the channel in relation to the devices and inlet/outlet. The area of Pt (S_{Pt}) immersed in the buffer with a conservative estimate at ~7.85× 10^6 μm^2 (defined by $\pi r \times L$=3.14×0.25×10×10^6 μm^2, here, r is the radius of Pt wire, L is the length of the microfluidic channel; half of the Pt wire was immersed in the buffer), which is nearly 2000 times larger than the area of graphene $S_{graphene}$ of 4.05× 10^3 μm^2 defined by the graphene channel length of 45 μm and width of 90 μm.

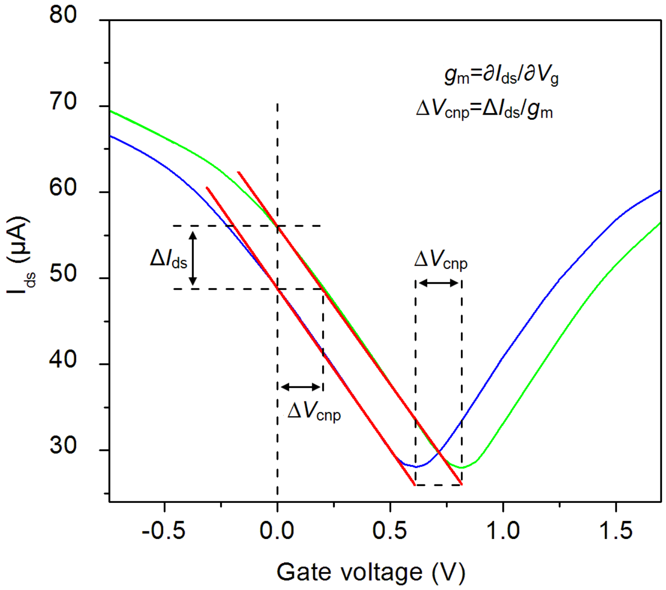

Supplementary Figure 9. The schematic diagram for determining the shift of I_{ds}-V_g transfer curve (or ΔV_{cnp}) by $\Delta I_{ds}/g_m$.

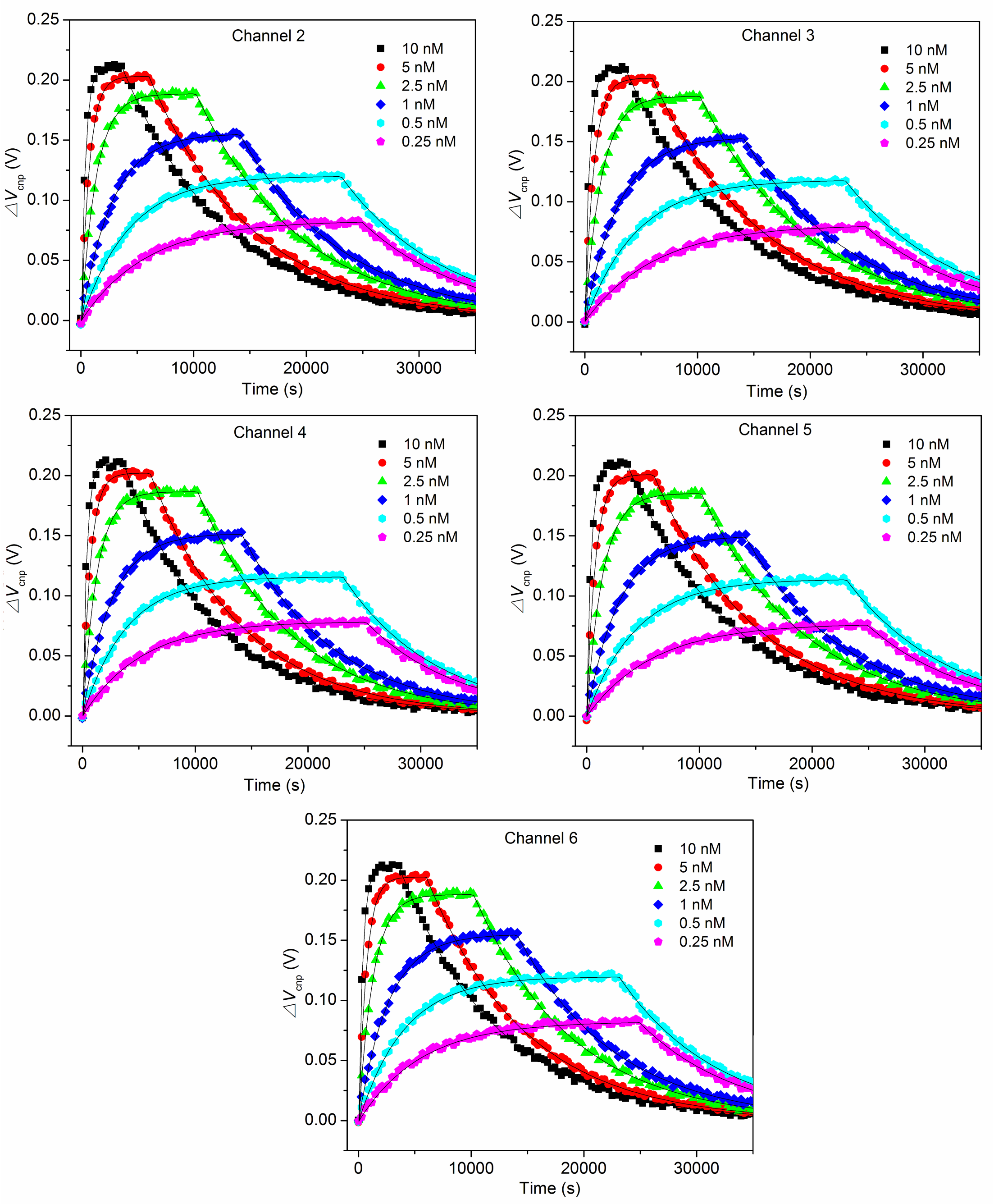

Supplementary Figure 10. Real-time sensorgrams of DNA-DNA (P20-T20) hybridization with different T20 DNA concentrations recorded in Channels 2-6.

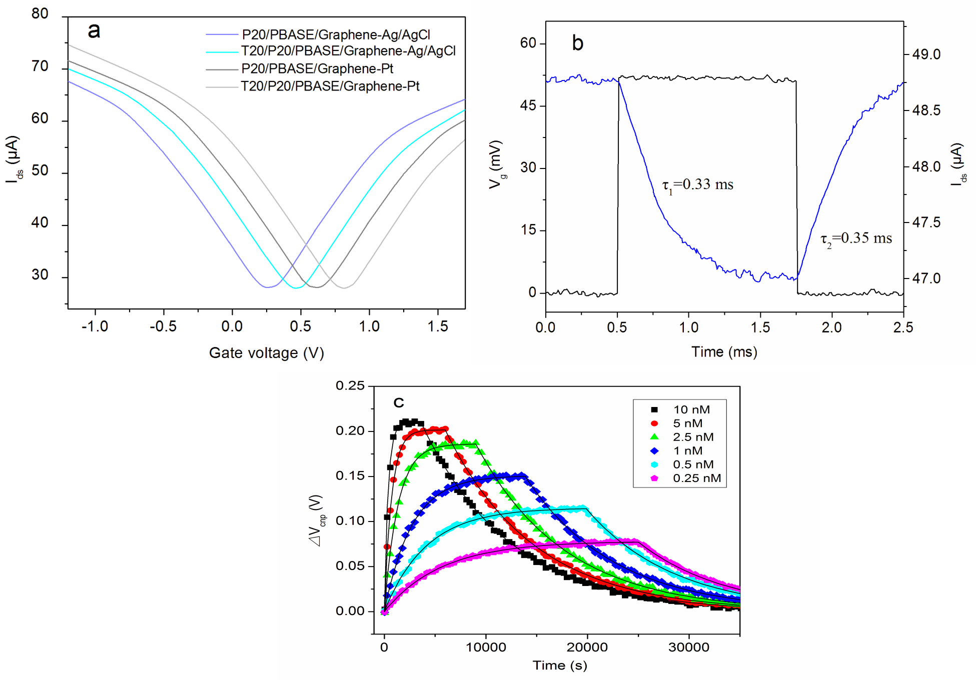

Supplementary Figure 11. The performance of G-FET device gated by using a non-polarizable Ag/AgCl electrode. a, Comparison of V_g-I_{ds} transfer curves of G-FETs using Ag/AgCl electrode and Pt electrode before and after target DNA adsorption. **b,** Transient response of I_{ds} to V_g pulsed from 0 to 50 mV. **c,** Real-time sensor responses to DNA hybridization and dissociation using the G-FET gated by a Ag/AgCl electrode.

To estimate the effect of polarizability of the Pt electrode on kinetic measurement, we performed the experiments with the same G-FET devices but gated using a non-polarizable Ag/AgCl electrode. Supplementary Figure 11a compares the transfer curves of G-FETs using Ag/AgCl and Pt electrodes, respectively, before and after target DNA adsorption. The Ag/AgCl electrode sets the solution potential about 300 mV lower than that of the Pt electrode. This difference can be attributed to the different solution-metal interfaces and different surface electrochemistry (Electrochemical Methods: Fundamentals and Applications, Wiley, New York, 2001). However, for both Ag/AgCl and Pt gated G-FETs, the shape of transfer curves are very similar, and the shift of transfer curves (or ΔV_{cnp}) is nearly identical after DNA hybridization. This result indicates that although the Pt electrode is polarizable, it produced little signal across the G-FET devices during DNA hybridization. Supplementary Figure 11b shows the transient response of I_{ds} to V_g pulsed from 0 to 50 mV. I_{ds} rapidly responses to V_g pulse with a characteristic fall and rise times at ~ 0.33 ms and 0.35 ms, respectively, similar to when the Pt electrode was employed (Supplementary Figure 3b). From the fit in Supplementary Figure 11c, we obtained the association rate constant, $k_a = 2.53\times 10^5$ M^{-1} s^{-1}, the dissociation rate constant, $k_d = 1.15\times 10^{-4}$ s^{-1} and the association equilibrium constant, $K_A = k_a/k_d = 2.20\times10^9$ M^{-1}. These results are in excellent agreement with those obtained by using a Pt electrode.

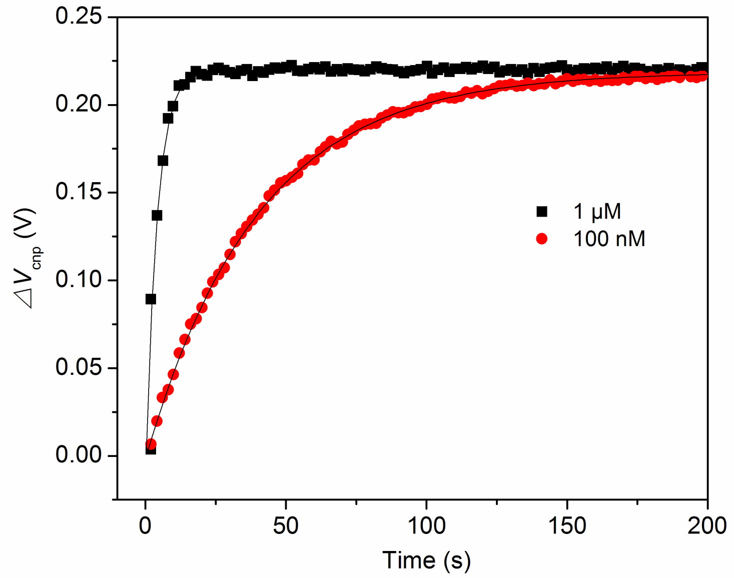

Supplementary Figure 12. Real-time sensorgram of DNA-DNA (P20-T20) hybridization with target DNA' s concentrations at 100 nM and 1 µM, respectively. The kinetic curves reach their respective equilibration in less than 3 minutes for 100 nM DNA and less than 1 minute for 1 µM DNA.

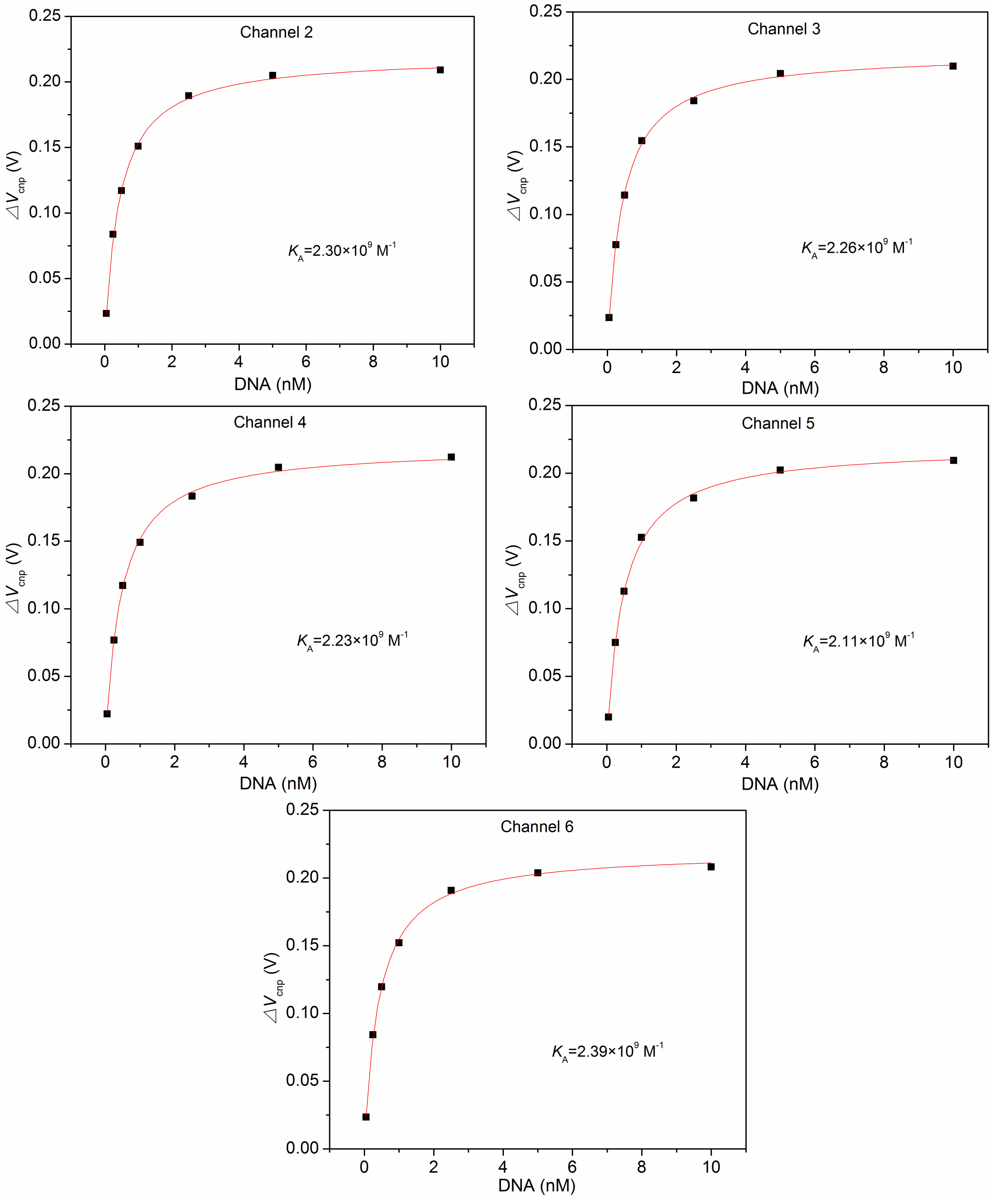

Supplementary Figure 13. Steady state responses of ΔV_{cnp} as a function of DNA concentrations in Channels 2, 3, 4, 5, and 6 in detection of T20-P20 hybridization.

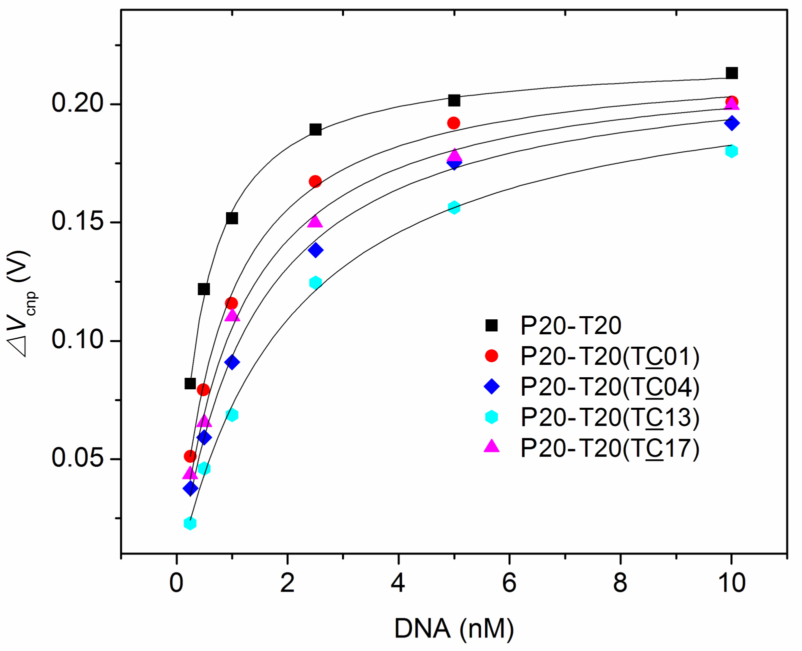

Supplementary Figure 14. Steady state response as a function of the target DNA concentration for interactions between P20 and its complementary sequence T20 as well as between P20 and the mutated sequences with one mismatched base pair at different locations T20(TC01), T20(TC04), T20(TC13) and T20(TC17) as labeled.

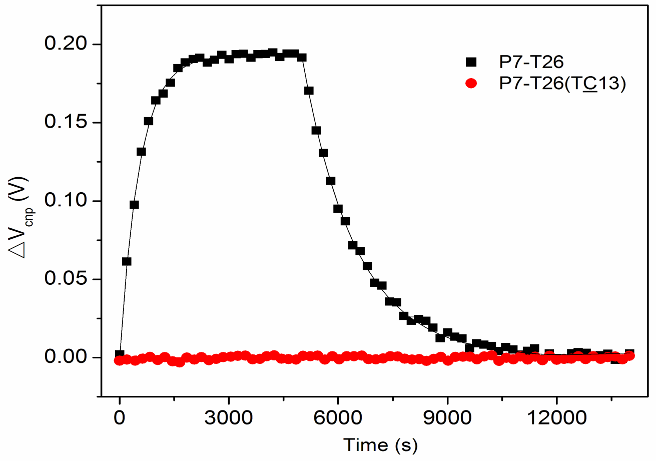

Supplementary Figure 15. Kinetic curves of hybridization of T26 and its mutant T26 (TC13) with P7. Kinetics of association and dissociation for P7-T26 hybridization can be measured but not for P7-T26 (TC13) hybridization. Both concentrations of T26 and T26 (TC13) are 5 nM.

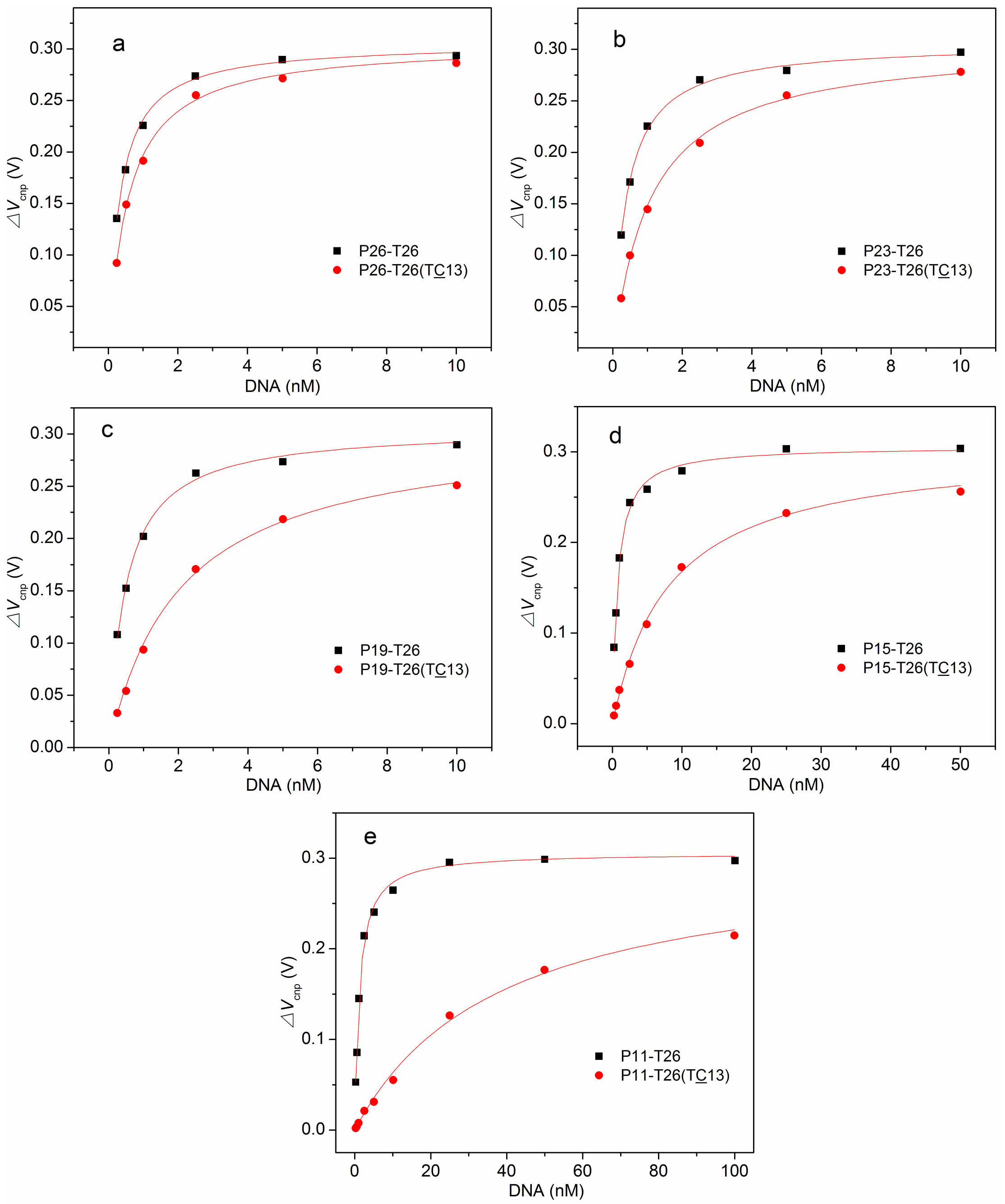

Supplementary Figure 16. Steady state response as a function of the target DNA concentration for detecting T26 and its mutant T26(T<u>C</u>13) with probes in different sizes. a, P26. d, P23. c, P19. d, P15. e, P11.

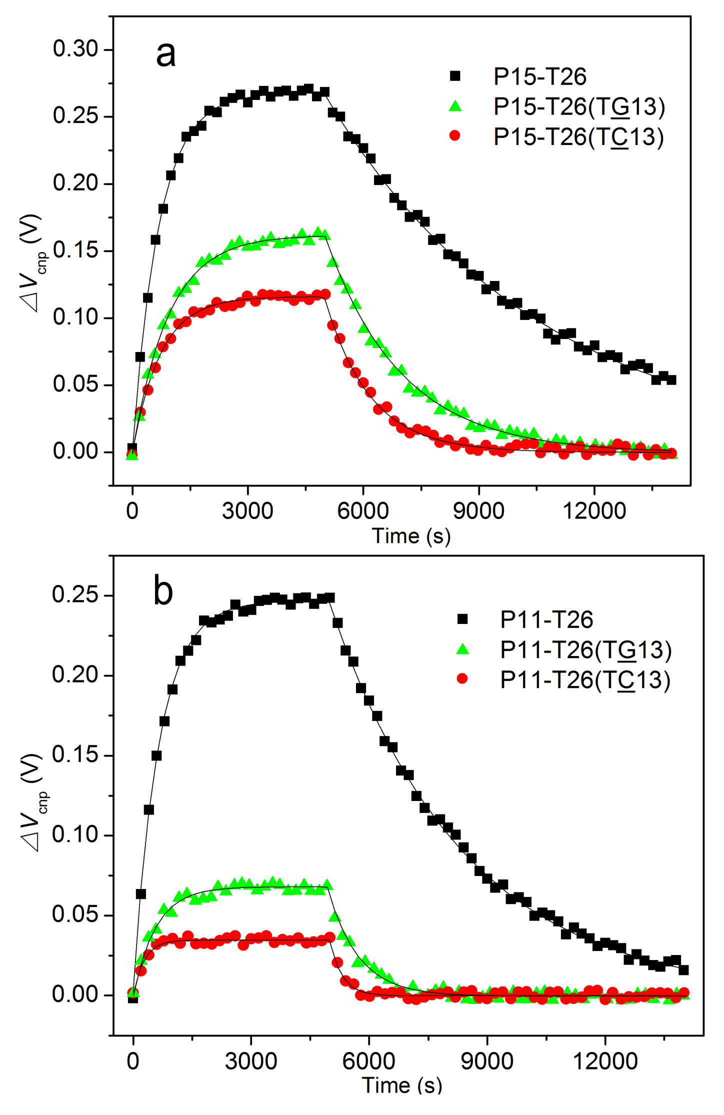

Supplementary Figure 17. Kinetic curves of hybridization between probes of two different sizes and targets of different mutations at the same sequence position. a, T26, T26(T<u>G</u>13) and T26(T<u>C</u>13) with probe DNA P15. b, T26, T26(T<u>G</u>13) and T26(T<u>C</u>13) with probe DNA P11. Both concentrations of T26(T<u>G</u>13) and T26(T<u>C</u>13) are 5 nM. The extracted affinity and kinetic constants from the curves for the figure above are listed in Supplementary Table 1.

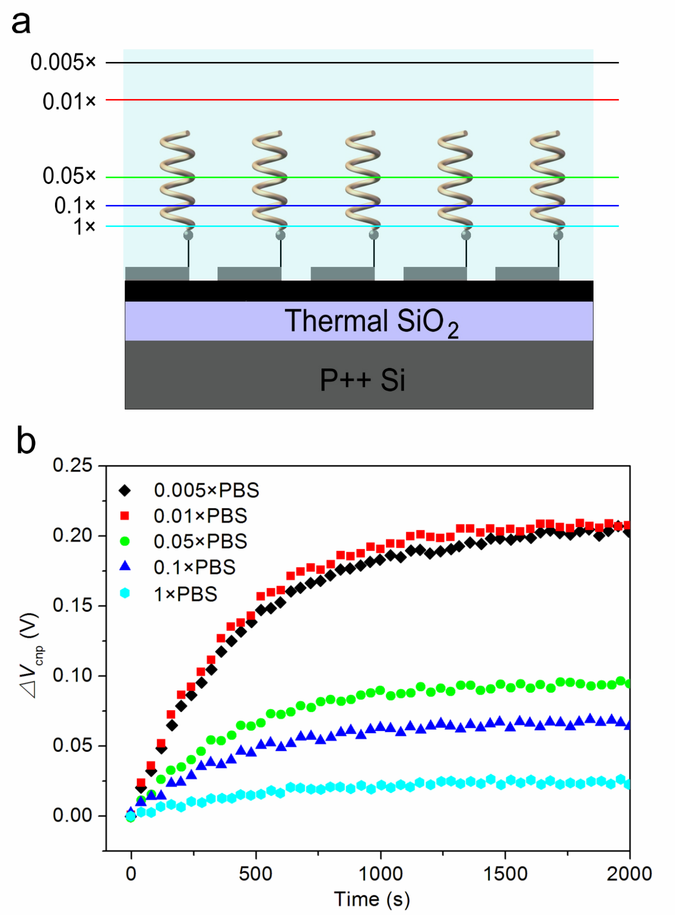

Supplementary Figure 18. Impact of the Debye screening on DNA sensing. a, Schematic (not to scale) diagram shows Debye length from the device surface. 1×, 0.1×, 0.05×, 0.01× and 0.005× lines represent Debye length values from the graphene surface in 1×, 0.1×, 0.05×, 0.01× and 0.005×PBS buffers, respectively. **b,** Real-time sensor response (ΔV_{cnp}) of P20-T20 hybridization in different buffers. Here, the concentration of T20 is 5 nM.

The ionic strength of the solution has a strong effect on device sensitivity for DNA detection. Here, the P20 functionalized G-FET device in the corresponding buffers were used as the reference. The maximum of sensor response ΔV_{cnp} increases as the ionic strength decreases from 1× PBS to 0.01× buffer. With further reduction of the ionic strength to 0.005× buffer, the maximum of ΔV_{cnp} did not change much. The ionic strength of 0.01× buffer yields a Debye length of ~7.3 nm, which is slightly larger than the height of the target DNA (T20) of ~6.8 nm. Thus, all charges on T20 are unscreened at the sensor surface, resulting in a significant response. However, for the 5-fold (0.05× PBS, Debye length ~3.3 nm), 10-fold (0.1× PBS, Debye length ~2.3 nm) and 100-fold stronger (1× PBS, Debye length~0.7 nm) buffers, the Debye lengths are significantly shorter and screen DNA's intrinsic charges partially, which led to low sensitivity of the sensor.

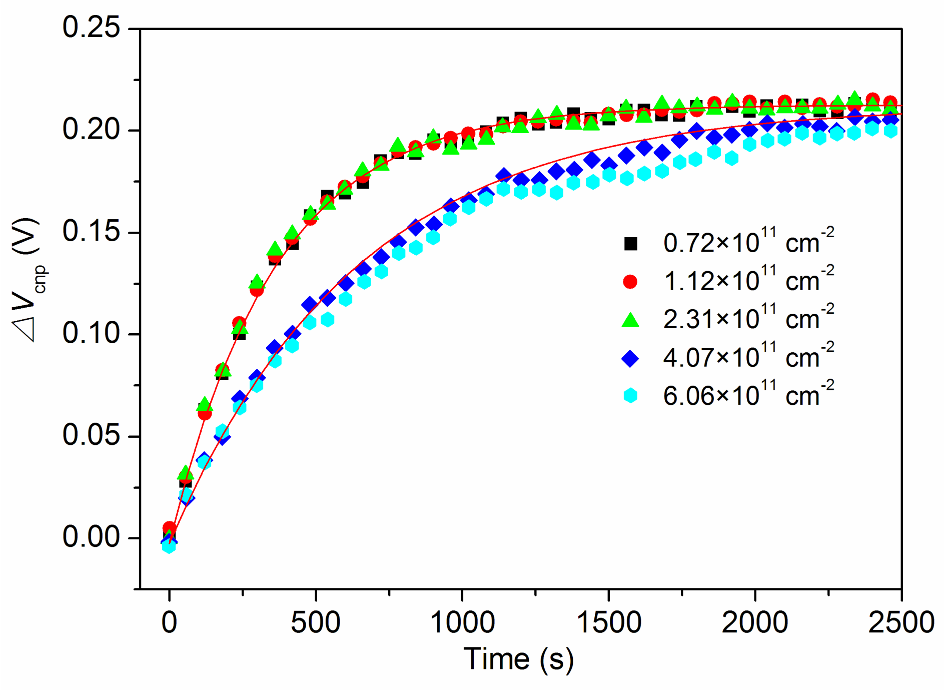

Supplementary Figure 19. DNA hybridization kinetics at different probe densities as labeled. DNA probe P20 at different immobilization density hybrids with its complementary DNA T20 at 10 nM concentration. The data shows slower target-capturing rates at higher (4.07×10^{11} and 6.06×10^{11} cm^{-2}) probe densities with poorer exponential fit than at lower (0.72×10^{11}, 1.12×10^{11} and 2.31×10^{11} cm^{-2}) probe densities. Here, all the probe densities were estimated by using the method described in the caption of **Supplementary Figure 8**.

Supplementary Table 1. Kinetic constants and binding affinities between probes of two different sizes (P15 and P11) and targets of different mutations at the same sequence position (T26, T26(TG13), T26(TC13)).

	k_a ($\times 10^5$ M^{-1} s^{-1})	k_d ($\times10^{-4}$ s^{-1})	K_A ($\times10^9$ M^{-1})
P15-T26	2.53	1.81	1.40
P15-T26(TG13)	1.11	5.07	0.22
P15-T26(TC13)	1.04	8.53	0.12
P11-T26	2.47	2.96	0.83
P11-T26(TG13)	0.85	12.82	0.067
P11-T26(TC13)	0.82	31.36	0.026
